# Data to support the modelling of integrated groundwater-surface water flow in permafrost regions, using the Carcajou Watershed, Northwest Territories, Canada

**DOI:** 10.1016/j.dib.2026.112802

**Published:** 2026-04-30

**Authors:** Melissa I. Bunn, Omar Khader, Eric Kessel, Steven K. Frey, Andre R. Erler, Hazen A. Russell

**Affiliations:** a Natural Resources Canada, Geological Survey of Canada, 601 Booth Street, Ottawa, Ontario K1A 0E8, Canada; b Aquanty Inc., 600 Weber St. N., Unit B, Waterloo, Ontario N2V1K4, Canada

**Keywords:** Hydrology, Hydrogeology, Climate change, Numerical model, Conceptual model, Finite element model

## Abstract

> 50 % of Canada's landmass is underlain by perennially frozen ground (permafrost). These permafrost regions are changing rapidly in response to a warming climate, with Canada’s arctic warming at a rate > 3 times higher than global average. As permafrost distribution shifts in response to this warming, it alters the water cycle, affecting both water quantity and water quality. However, large-scale hydrological models that integrate climate change impacts often lack permafrost representation.

The Carcajou Regional Model Dataset contains the inputs needed to construct a standalone groundwater model or a fully integrated groundwater – surface water model of the Carcajou watershed in the Northwest Territories, Canada, a zone of transition between continuous permafrost and discontinuous permafrost. The three-dimensional geological data was created by refining national-scale mapping products, with local-scale data. Land cover, topography, and surface water flow were generated from national scale mapping products. Climate forcing data for the period from 2010 to 2016 was generated based national scale historical products.

The proper approach to representing permafrost in large-scale hydrological models is largely undefined, with no consensus on conceptualization or parameterization. This dataset allows researchers to rapidly test new approaches to permafrost representation, without the need for additional data collection. The data set includes long term surface water discharge records to assess model performance and compare representations. The data set can be further adapted to simulate projected permafrost distribution scenarios and be used to forecast the effect of permafrost change on water quantity.

Specifications Table

**Table:** 

Subject	Hydrology and Water Quality or Water Science and Technology
Specific subject area	Integrated groundwater – surface water modelling at regional scales with permafrost representation
Type of data	Processed – vectors (.shp format), rasters (ArcInfo ASCII Grids), tables (delimited text/ASCII), HydroGeoSphere model commands (text/ascii)
Data collection	The Carcajou Watershed Boundary was downloaded from Canada’s National Hydrometric Network (Watershed 10KB000). The watershed outline was simplified using manual GIS editing tools. The classified surface water network, digital elevation model (DEM), liquid water flux (LWF), potential evapotranspiration (PET), landcover, leaf area index, soil temperature, and parameterized soil, surficial, and bedrock geology data sets extracted for the model domain from available national scale data sets [1]. The available surface water network was filtered so that Strahler order 1 and 2 streams were excluded. This filtering reduced the model size to facilitate efficient simulations runs. The available national scale DEM is void filled, canopy corrected and includes lake bathymetry. This elevation model included in this dataset was hydroconditioned by rasterizing the filtered stream network and burning this into the national scale DEM. The available liquid water flux data is the sum of liquid precipitation and snowmelt extracted from a national scale data set of monthly normals [1] Snowmelt calculated as the sum of daily changes in snow water equivalent (SWE) from the ERA5-Land dataset. The available potential evapotranspiration was calculated based on the Hogg Method [2], and uses the maximum and minimum temperatures from [1]. The available land cover is from the North American Land Change Monitoring System (NALCMS) Land Cover 2015 v2. The data set from which it was extracted was reprojected to match the projection of the model. The available leaf area index dataset was derived from the ESA Copernicus Global Land Operation Leaf Area Index. The available soil temperature data set is a monthly averaged data set, bias corrected based on random forest regression based on ERA5-Land soil temperature estimates, along with information about the seasonal cycle, ERA5-Land 2 m air temperature, the ESA-CCI land cover type, elevation (from the Copernicus DSM), ESA-CCI snow water equivalent (SWE), and geospatial coordinates. The bedrock geology and quaternary geology datasets were extracted from published maps [3,4]. The soils geology was extracted from a national scale data set that includes peatlands and bedrock outcrops [1]. The elevation of the bedrock surface was derived from the mapping presented in [5]. Where this mapping did not provide complete coverage of the model, the methods used in [5] were combined with the quaternary geological mapping [4] and point data from [6] to compvlete the coverage. The model Finite Element Mesh was constructed using the Algomesh software package [7], using the stream network dataset to locally refine the mesh near surface water features.
Data source location	Carcajou Watershed, Northwest Territories, Canada
Data accessibility	Repository name: Federal Geoscience Platform Data identification number: https://doi.org/10.23687/0ae8eac8-c651-b94d-a6c5–2475694ddf7c Direct URL to data: https://ftp.maps.canada.ca/pub/nrcan_rncan/Geology_Geologie/groundwater-eau_souterraine/crw_model-modele_crw/model/CRW.7z
Related research article	

## 1 Value of the Data

• This dataset provides all the data needed to construct a standalone groundwater model or a fully integrated groundwater – surface water model of the Carcajou watershed in the Northwest Territories, Canada.
• This dataset is a regional scale integrated groundwater-surface water model that includes permafrost, this type of integrated model has not been previously available at regional scales.
• This dataset is flexible, containing a limited number of software specific files, to allow for application to any commonly used hydrological or hydrogeological modelling software for broader application.
• This dataset can be used to rapidly construct models that allow testing of different permafrost conceptual models, or parametrization for large scale models
• This dataset be used to construct models that permit the assessment of the impact of future permafrost distributions on surface water flow.

## 2 Background

Due to challenges with access, and a sparse monitoring network, the understanding of regional groundwater flow and the interaction between groundwater and surface water is limited in regions underlain by permafrost. With temperatures rising in Canada’s arctic at rates more than three times higher than the global average [8], large-scale models can be a valuable tool to assess the associated changes to water cycle dynamics and to plan for adaptation [9]. As work to develop national scale integrated groundwater – surface water models for Canada is ongoing [1] and >50 % of Canada’s landmass is underlain by permafrost [10], the objective of this data release is to provide a model capable of testing approaches for representing permafrost in regional-scale groundwater – surface water models. The Carcajou watershed in the Northwest Territories was selected for this purpose as is located within the transition from continuous to discontinuous permafrost, and has a long term, (2006 to 2023) year-round surface water gauging station. The thin veneer of surficial materials, and high variation in elevation (2312 mASL to 25 mASL) within this watershed are features similar to many watersheds within the Canadian north. This dataset for the Carcajou watershed can be applied to assess permafrost representation in regional-scale models.

## 3 Data Description

The main data folder contains three subfolders: Carcajou_Model; CRCW001-Coarse; and transient-daily.

The Carcajou_Model folder contains many of the model input files needed to run this model or construct a model within a different software package. Its structure is described below. The CRCW001-Coarse folder contains the HydroGeoSphere instruction files used to run the model within the software, its contents are described below. The transient_daily folder contains the climate forcing data for the model runs, as described below.

The Carcajou_Model folder contains 18 sub folders as follows:

Bedrock – This folder contains three raster files with bedrock lithology data for the upper bedrock (Upper 10 m) (carcajou_brock_dissolved_esri102008_v3.asc) (carcajou_brock_dissolved_esri102008_bot_v3.asc), and the middle bedrock (From 10 m below the bedrock surface to 30 m below ground surface), and deep bedrock (carcajou_brock_dissolved_esri102008_botm2_v3.asc) (from 30 m below ground surface to – 50 mASL)
Channels – this folder contains the file with .inc extension which is an ascii text file in HGS format which lists groups of nodes that denote surface water channels, and the channel that they are to be assigned to. The file with the suffix cprops is an ascii text file which contains the HGS formatted list of channel properties.
Etprops – Contains input files for the evapotranspiration calculations. The file with the .inc suffix is an ascii text file in HGS format that contains instructions to select mesh faces based on the land cover raster and assign representative properties. The file with the etprops suffix contains the HGS formatted evapotranspiration properties file. The subfolder LAI contains .inc files which are delimited text files with a time series of the leaf area index. The LAI folder contains files with the .inc suffix for each of the landcover types. These files are delimited text files containing leaf area index values for each model timestep for the major landcover types.
Forcing – This folder contains two delimited text files with the .inc suffix, these are the HGS formatted instruction files for the Liquid Water Flux (liqwatflx.inc), and the potential evapotranspiration (pet_era5.inc) which contain a list of raster files to read for each of the model timesteps
Grid – This folder contains the AlgoMesh formatted project file that can be used to modify the model mesh (Caracajou_Base_Coarse_simp250m_optimize6_30000nodes.amproj), and the AlgoMesh output for ingestion into the HGS 3D grid building process, for vertical mesh modification (Caracajou_simp250m_optimize6_30knodes.ah2). Also included are the HGS instruction files to construct vertical layers. In the distribution version of the .grok file, the previously built HGS mesh is used, and this folder is not called.
Kmaps – This folder contains ascii raster files which are maps of the saturated hydraulic conductivity distribution of the upper 1 m of the model, with separate files for mineral soil (Ksat_pedoTrans_1m_102,008.asc), peat from 0 to 0.1 m (Ksat_pedoTrans_Peat_0.1m_102,008.asc), and peat from 0.1 m to 1 m (Ksat_pedoTrans_Peat_1m_102,008.asc).
Landcover – This folder contains a file with the .inc suffix which is an HGS formatted ascii text file with instructions for selecting landcover zones and assigning the associated runoff properties. The file with the .oprops file is an HGS formatted ascii text file containing the surface runoff properties, and the raster “nalcms_2015_v2_esri102008.asc” contains the landcover zones.
Node_list – The folder contains files with the .inc suffix which is an HGS formatted instruction file that calls text files to assign surface water observation locations. The files with the suffix .txt are lists of notes within the numerical mesh at which surface water flows are calculated, coincident with Water Survey of Canada gauging stations.
Peatland – Contains an HGS formatted output file from AlgoMesh (carcajou_PeatLand_17 %OrganicCarbon.echos) containing the list of elements contained within mapped peatland areas based on the file (carcajou_PeatLand_17 %OrganicCarbon_102,008.shp). The AlgoMesh project file (Caracajou_Base_Coarse_simp250m_optimize6_30000nodes_peat.amproj) was used to generate this file. Also included are HGS formatted instruction files to assign the peatland elements.
Permafrost – Contains a HGS formatted mesh element selection file (carcajou_main_streams_1000buffer_31Kmexh.echos) which selects the elements within a 1 km buffer around streams of Strahler order 6 and 7 or greater to a depth of 20 m below the top of bedrock represent taliks below major watercourses.
Rill_modify – This folder contains a text file with a suffix .dat which is a HGS formatted table listing element numbers and a rill storage value.
Soil – Contains rasters for each soil layer (0, 10, 100 cm) in the naming format depth_carcajou_ESRI_….asc. Each raster contains soil maps used to assign HGS zone numbers. These zone numbers are then used to assign subsurface properties using the .inc files in the “subsurface” folder.
Subsurface – This folder contains HGS formatted instruction files in ascii test that provide instructions for the selection of numerical elements to assign properties (BRock_surficial_N_v3.inc); a HGS formatted instruction file to assign each group of elements to a hydrostratigraphic unit and read its properties (subsurface_assignment_v3.inc); and to assign hydraulic conductivity to soil units based on the raster in the kmaps folder. The folder also contains a HGS formatted properties file in ascii text (soil.mprops) with the hydraulic parameters for each hydrostratigraphic unit.
Surfaces – This folder contains two raster files in ascii format which contain the surface elevation data for the ground surface (c1w_hrdem_voidfillaw3d30_carcajou_w127_w130_n64_n65_ESRI102008.asc), and top of bedrock surface (Carcajou_BR_HRDEM_102,008.asc).
Surficial – This folder contains the surficial geology maps as ascii rasters with the various geological units represented by a zone number. (fultonSG_carcajou_ESRI102008_final_2.asc, fultonsg_carcajou_esri102008_final_2v3.asc, and fultonsg_carcajou_esri102008_final_2botv3.asc. These rasters are then used to assign subsurface properties using the .inc files in the “subsurface” folder.
Temp – This folder contains the C1W subfolder along with the HGS formatted ascii text instruction file soil_freezing.inc which provides the parameters for the soil freezing representation within HGS

The C1W subfolder contains the transient_monthly folder along with the HGS formatted delimited text files listing a model timestep and the associated soil temperature raster. The instruction file soil_freezing.inc which provides the parameters for the soil freezing representation within HGS

The transient_monthly folder contains a series of ascii raster files with monthly average soil temperature maps for each month from January 1981 to August 2020. The naming convention is c1w_tsl#_ccj1_yyyymm.asc where the # is associated with a soil depth level as in the experimental design section.

The climate forcing data for the project (within the transient_daily folder) are a series of rasters trimmed to a rectangular box with bounds of −1618,000 to 3058,000 m east, −1508,000 to 3223,000 m north in Albers Equal Area coordinates, at a resolution of 5 km x 5 km encompassing the model domain. These rasters are trimmed from the climate forcing data used in the Canada1Water project (Canada1Water, 2025). The naming convention for the files is as follows “era5_pet_era5_ccj1_yyyymmdd.asc” for the potential evapotranspiration data, and “era5_liqwatflx_ccj1_yyyymmdd.asc” for the liquid water flux data. There is one raster for each parameter for each day between January 2, 1981, to August 31, 2020. Units are kg/m^2^/day for both variables.

The instructions for running the HGS model and calling all the previously discussed files are located within the CRC001-Coarse folder. The “CRW.grok” file is the main instruction file used to build the model within HGS based on the input files. The CRW.output_variable.control contains the instructions for what model output should be produced while the model is running. The CRW.plot.control file contains the post-processing instructions for formatting the output for visualization software. The “IC” subfolder contains 3 files with the prefix CRWo.head_ that contain the pseudo-steady state channel conditions (“…chan.0028”), overland flow conditions (…old.0028), and porous media condition (…pm.0028). These are read by HGS to provide initial conditions for the start of the simulation.

## 4 Experimental Design, Materials and Methods

**Fig. 1:**
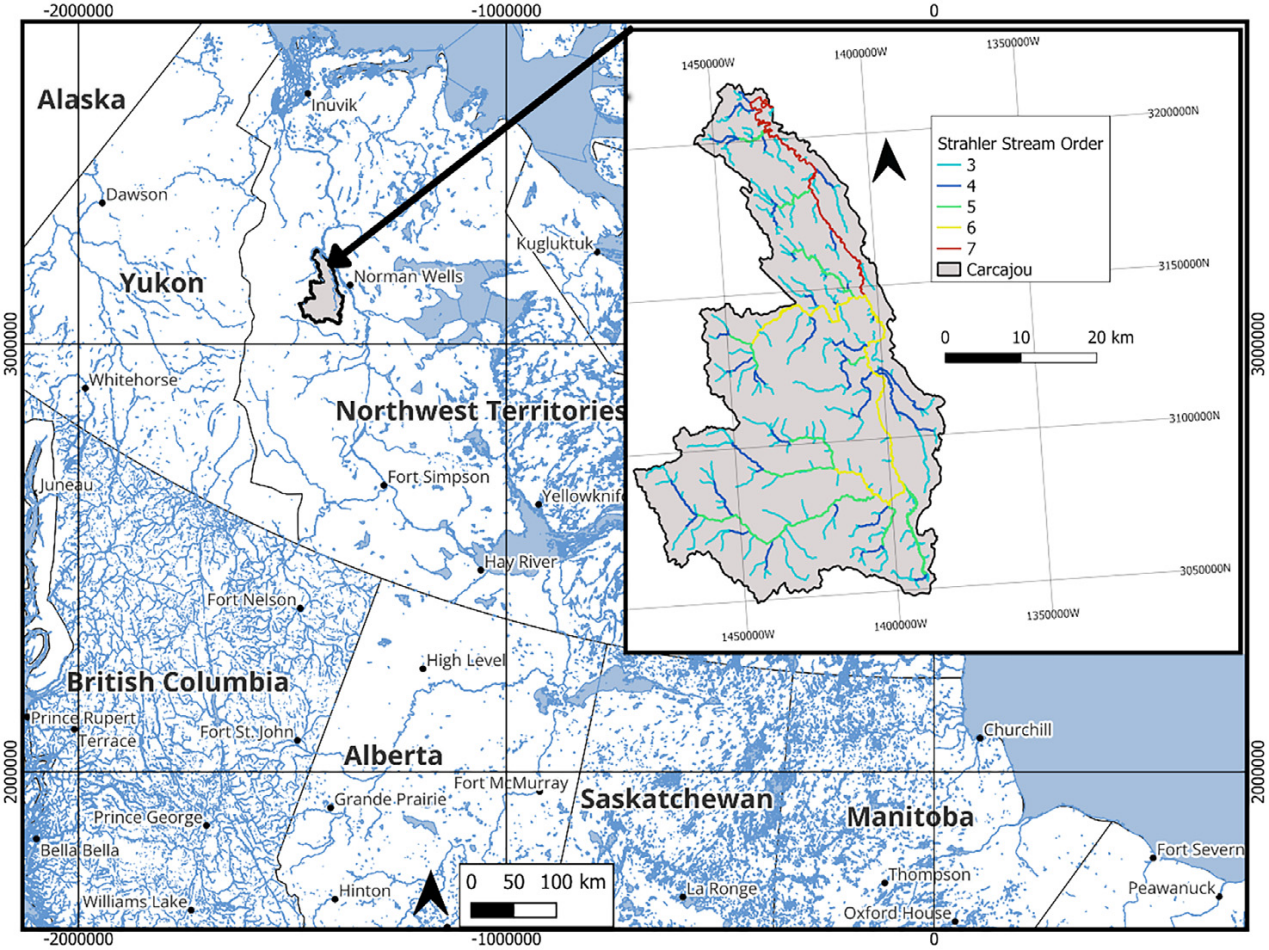
Watershed location and stream network.

The model domain is 7200 km^2^ and covers the Carcajou Watershed in the Northwest Territories (shown on Fig. 1). Model boundaries were derived from the National Hydro Network (NHN) [11] (10kb000). The stream network available through the Canada1Water project [1]was used to delineate surface water flow channels as it includes harmonized Strahler stream order as shown on Fig. 1.

The model domain and stream network were used to constrain the construction of the finite element mesh (FEM). The software Algomesh [7] was used to create the FEM which discretely resolves Strahler Order 3 and greater streams as 1-dimensional channels in the model. There is a 250 m model resolution along the channel network and up to 1000 m away from channels. There are 30,960 FEM nodes per sheet and the subsurface component of the model was vertically discretized into 7 layers (8 sheets) resulting in a total of 247,680 nodes for the subsurface domain. The subsurface layers were implemented as follows:

• Layer 1: This is the lower bedrock unit from 30 m below surface to – 50 m above sea level. This layer represents the Precambrian basement rock, and hydraulic properties are provided in Table 1. The values are mainly derived from textbook values for representative geology that were modified during the calibration process. This unit is sub-permafrost and does not freeze.

**Table 1:** Model zonation and hydrostratigraphic parameterization.

Layer	Zones	Horizontal Hydraulic Conductivity (m/s)	Hydraulic Conductivity Anisotropy (Vertical/Horizontal)	Residual Saturation
Bedrock	Precambrian	1.02E-08	0.10	0.098
	Fractured Precambrian	1.00E-06	0.10	0.098
	Cambrian Aquifer (Shale and Dolostone)	6.97E-06	0.10	0.098
	Devonian - Cambrian Aquifer (Arenite, dolostone and shale)	2.40E-07	0.10	0.098
	Devonian, and Silurian Aquitard (Shale and Dolostone)	8.62E-09	0.10	0.098
	Lower Cretaceous Shale	1.33E-09	0.04	0.098
Quaternary	Glaciofluvial Sand and Gravel	2.33E-04	0.10	0.056
	Till	5.00E-06	0.10	0.085
	Bedrock Outcrop	6.30E-06	0.10	0.106
	Alluvial	1.49E-06	0.10	0.045
	Silty Clay Loam	1.29E-05	0.10	0.090
	Colluvial	1.49E-06	0.10	0.045
	Clay Loam	9.08E-04	0.10	0.065
	Clay	2.98E-04	0.10	0.085
	Sandy Clay Loam	1.49E-03	0.10	0.059
	Sandy Loam	3.94E-03	0.10	0.050
	Silt Loam	3.93E-03	0.10	0.066
	Loam	1.41E-03	0.10	0.059
Organic	Peat	2.80E-04	1	0.040
• Layer 2: Is the middle bedrock from 10 m below the top of bedrock surface to 30 m below the top of bedrock surface. Bedrock units were mapped according to [3], and hydraulic parameters are provided in Table 1. Mapped bedrock is illustrated on Fig. 2. These units are perennially frozen, and hydraulic conductivity is adjusted using the following equation:
  $$K_{Frzone}=\;K_{sat}\;x\;10^{-Impedance\;Factor\;x\;Sw}$$

**Fig. 2:**
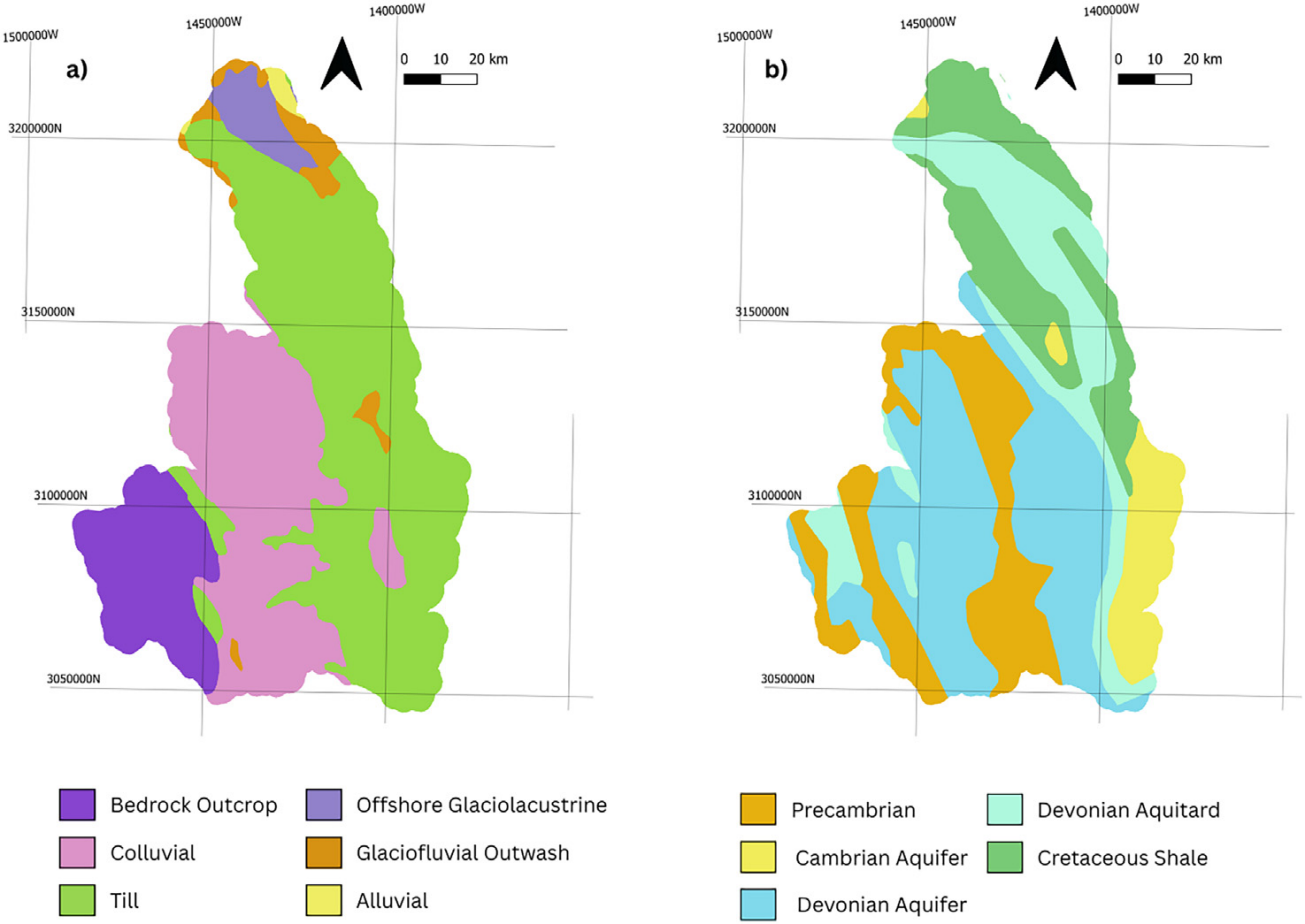
Quaternary (a) and Bedrock (b) geological material distribution datasets.

*Equation 1: Impedance Factor for Hydraulic Conductivity* [12] with an impedance factor of 1.4. Where$\;K_{sat}\;$is the saturated hydraulic conductivity, and Sw is soil water content This approximation is based on [13] and accounts for variably saturated conditions.

**Fig. 3:**
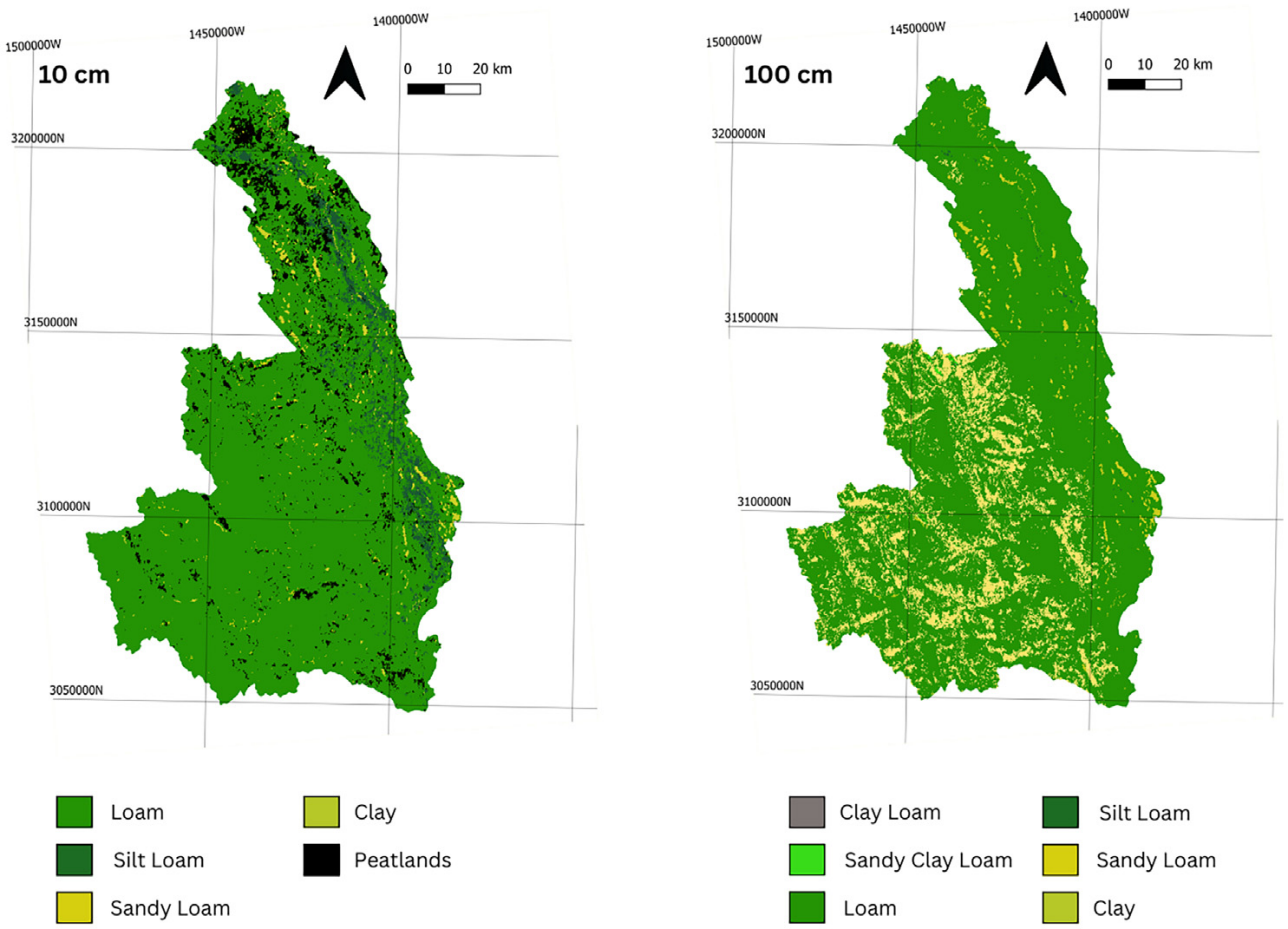
Mineral soil and peat distribution datasets for Layer 7 (10 cm) and Layer 6 (100 cm).

• Layer 3: Is the upper 10 m of the Bedrock Units: The bedrock surface was constructed based on the drift isopach map from [5]. Where the model domain did not intersect that map, the method from [5] was used, assigning random points at 500 m spacing and assigning drift thickness based on quaternary geology mapping [4]. To expand the approach of [5] to this watershed, a drift thickness of 5 m was added to thermokarst sensitive fluvial units. These drift isopach points were combined with depth to bedrock points from [6], and kriging was applied to generate a continuous isopach. This isopach was then subtracted from the DEM to produce the top of bedrock surface. Bedrock units were mapped according to [3] and hydraulic parameters are provided in Table 1. Below the permafrost table (2 m depth) these units are perennially frozen, and hydraulic conductivity is adjusted using Equation 1, with an impedance factor of 1.4.
• Layer 4: Is the lower Quaternary unit that extends from 1 m below the base of soil to the top of bedrock. The distribution of materials within this unit are based on [4], and hydraulic parameters are shown in Table 1. This layer is perennially frozen (permafrost), and the hydraulic properties are assigned using an impedance factor of 1.4 on the saturated hydraulic conductivities provided in Table 1 as shown in Equation 1. The mapped Quaternary units are illustrated on Fig. 2.
• Layers 5: Is the upper 1 m of the Quaternary unit. The distribution of materials within this unit are based on [4], and hydraulic parameters are shown in Table 1. This layer is seasonally frozen.
• Layers 7 and 6: Are soil layers, extending from surface to 0.5 m, and 0.5 m to 1 m depth from the DEM. The DEM is the bathymetry corrected and adjusted digital elevation model constructed as part of the Canada1Water Model. It is based on a void filled ArcticDEM/HRDEM with hydrological conditioning to implement watercourses [1]. The soil maps for these layers are derived from the Canada1Water dataset and are a combination of machine learning soil maps, peatland maps, peatland machine learning products, and mapped bedrock outcrops from surficial geology, and landcover maps[1]. Hydraulic properties were assigned using Rosetta V3 [14] for mineral soils, a fractured rock literature value for bedrock outcrops, and a logarithmic decrease with depth based on literature values for peat [1]. These layers are seasonally frozen (active layer). Mapped mineral soils and peatlands are illustrated on Fig. 3.

**Fig. 4:**
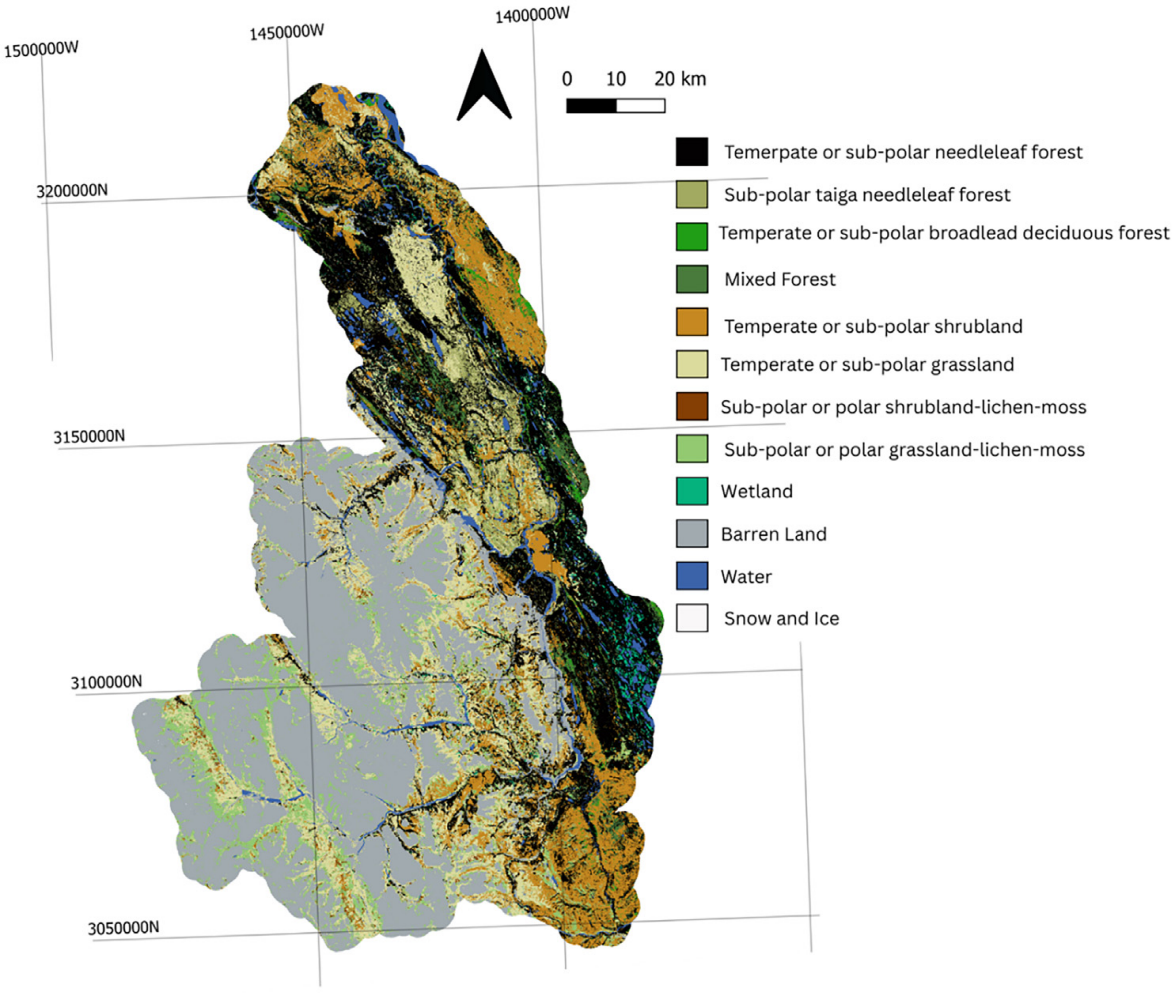
Landcover dataset.

Landcover within the model was obtained from the North America Land Change Monitoring System [15].The watershed is primarily barren land (31 %), temperate or sub polar needleleaf forest (21 %), and temperate or sub polar grassland (16 %). The remaining landcover is comprised of lichen-moss, tiaga needleleaf forest, mixed forest, and water, each at <10 %. Landcover is illustrated on Fig. 4.

**Fig. 5:**
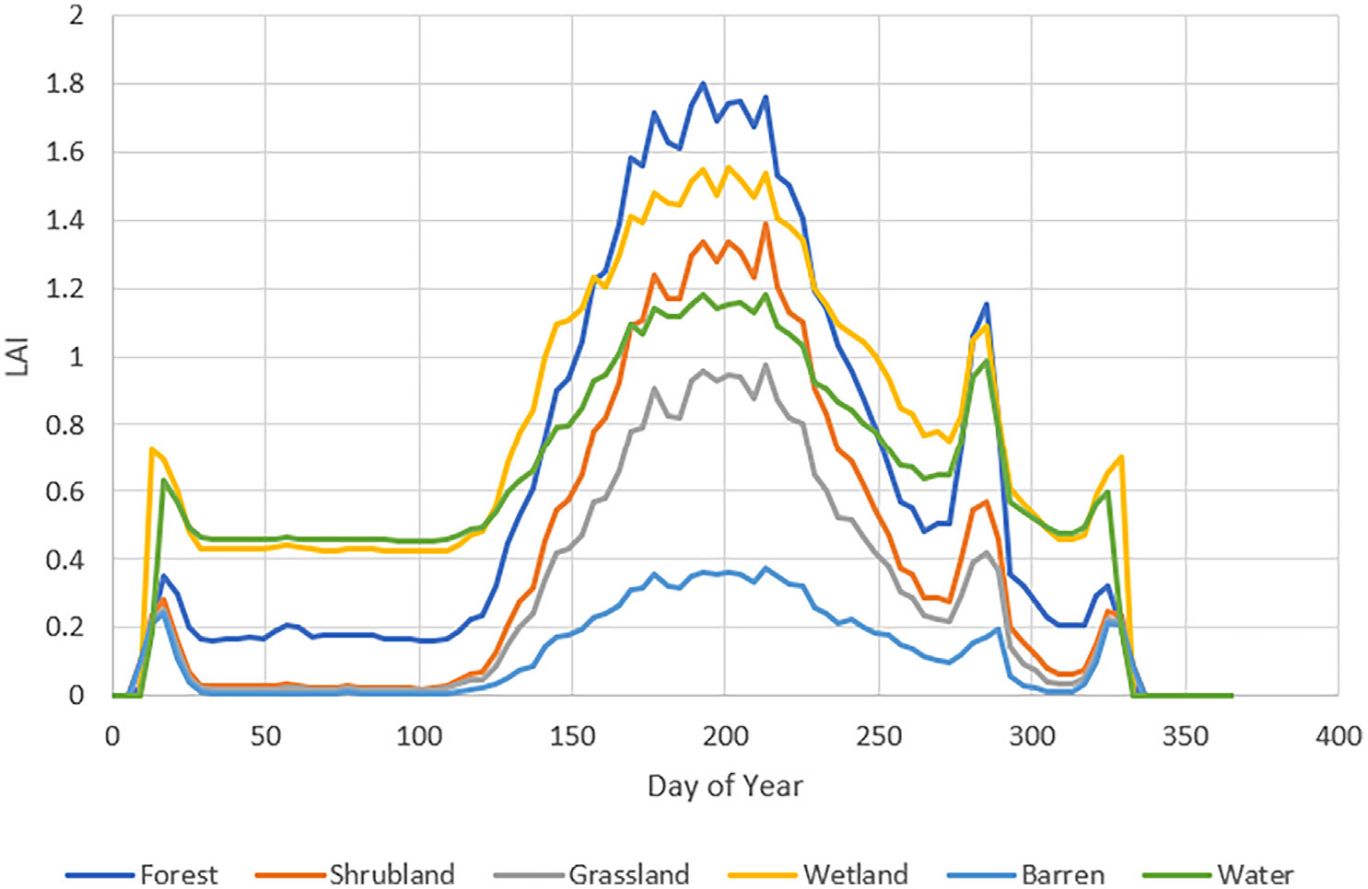
LAI time series datasets.

Plant transpiration is included in the Carcajou model and is calculated based on the leaf area index (LAI). LAI was assigned based on a remote sensing product from MODIS [16] with a 500 m resolution. The data was aggregated according to land cover and then decomposed into individual average seasonal condition time series for input. Average annual LAI time series for each landcover class in the model is illustrated on Fig. 5.

Time series grids of temperature and precipitation data were obtained from the Canada1Water Project [1]. Time series grids of snow water equivalent (SWE) were derived from the ERA5-land surface reanalysis product [17]. As HGS does not fully represent surface processes such as snow melt, liquid water flux (LWF) is calculated. LWF is the total volume of unfrozen water available to enter the groundwater system, runoff, or be evaporated. LWF is calculated according to Equation 2.

LWF=Precipitation−ΔSWE

*Equation 2. Liquid Water Flux Calculation*

**Fig. 6:**
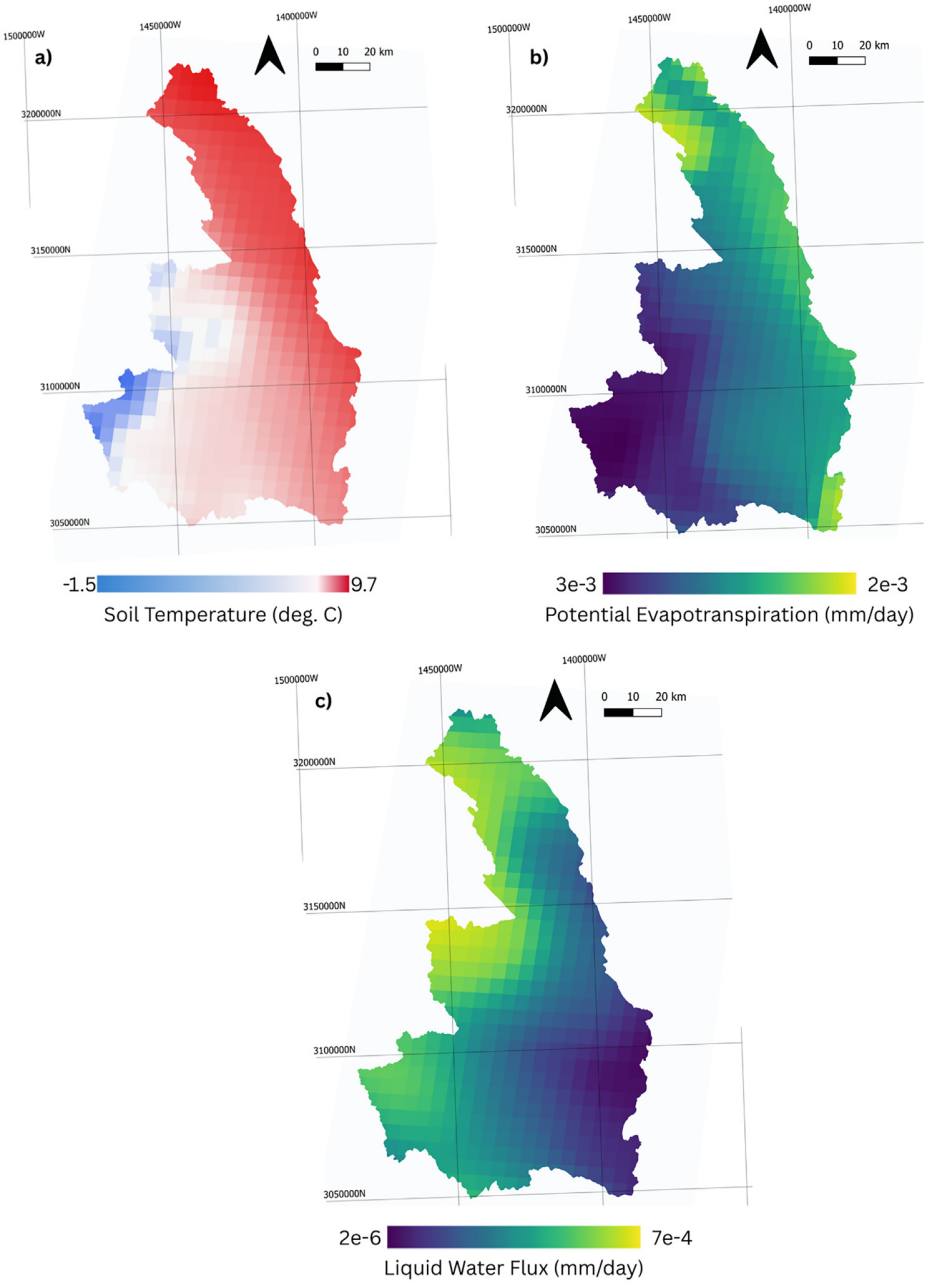
Climate forcing raster examples for June 6, 2017 (a) Soil Temperature from 0 to 0.1 m depth, (b) Potential Evapotranspiration, (c) Liquid Water Flux).

Potential evapotranspiration (PET) is used within HGS to calculate actual evapotranspiration (AET). Within the Carcajou model PET is represented using the Hogg method[2]. This method was selected based analysis conducted other large-scale models in the Canadian landmass[1]. Near surface air temperature, precipitation, PET, and SWE data for the Canadian land mass used in this model are illustrated on Fig. 6.

Soil temperature rasters are used to determine active layer freezing and thawing based on three depths intervals: 0 to 50 cm depth (the upper soil layer), 50 to 100 cm depth (the lower soil layer), and 100 to 200 cm depth (the upper quaternary layer). The soil temperature datasets are based on bias-corrected monthly average, random forest regression-based datasets [1]. They are presented as 0 °C threshold rasters. Active layer soil freezing is represented using the same impedance factor equation used for permafrost calculations.

One-dimensional surface water flow channels were implemented for all streams Strahler order 3 or greater. Numerical mesh nodes coincide with the center line of the channel. Channel properties (Channel width, friction, rill storage, streambed thickness, streambed hydraulic conductivity, bank height, incision depth) were assigned based on stream Strahler order. Channel width and friction were assigned using satellite image visual analysis. Stream bed material was assigned as silty soil. Values were assigned based on [18].

Evaporation parameters (depth, root depth, limiting pressures, canopy and interception storage), used to calculate actual evaporation from PET, varied with land cover type (e.g. forest, grassland, wetlands, and barren land). Values were assigned based on [19].

Overland flow parameters (manning coefficients, rill storage, length over which surface is coupled to subsurface) varied with landcover and were assigned based on [18]. then modified during the calibration stage.

In addition to the landcover based rill storage implemented within the overland flow parameters; an additional rill storage value was assigned to each surface element to represent potential depression storage at scales smaller than the numerical grid. These values were assigned per mesh cell based on DEM analysis [18].

Stream bed materials, evaporation parameters, overland flow parameters, and rill storage were adjusted during model construction to calibrate the model.

## Limitations

Data support is limited in northern Canada. The datasets provided herein were based on sparse in situ measurements, conceptual modelling, and where possible, bias correction approaches. The applied quaternary and bedrock mapping were both at a scale of 1:5000,000, which may miss smaller scale components of the geological system that can have larger influence on groundwater flow, an accepted limitation of this scale of modelling. Bias correction for the ground temperature data was based on a combined North American and Eurasian dataset for which no data was available within this watershed, and sparse data in the Canadian arctic in general. Similar data sparsity issues apply in the SWE dataset, for which spatial heterogeneity can be high at smaller scales. The precipitation and temperature datasets have well documented limitations, a component of which is also related to data sparsity [1].

## Ethics Statement

The Authors have read and follow the ethical requirements for publication in Data in Brief and confirm that the current work does not involve human subjects, animal experiments, or any data collected from social media platforms.

## CRediT Author Statement

**Melissa I. Bunn:** Conceptualization, funding acquisition, data curation, writing – original draft, validation, Project Administration; **Omar Khader:** Methodology, Software, data curation, visualization, processing, Validation, Investigation, Writing – Reviewing and Editing; **Eric Kessel:** Data curation, methodology; **Steven K. Frey:** Conceptualization, methodology, Writing – Reviewing and Editing, Supervision; **Andre R. Erler:** Data curation, methodology; **Hazen A. Russell**: Conceptualization, methodology, Writing – Reviewing and Editing
